# Evaluation of Antidiabetic Activity and Associated Toxicity of *Artemisia afra* Aqueous Extract in Wistar Rats

**DOI:** 10.1155/2013/929074

**Published:** 2013-06-04

**Authors:** Taofik O. Sunmonu, Anthony J. Afolayan

**Affiliations:** Research Center for Phytomedicine, Department of Botany, University of Fort Hare, Alice 5700, South Africa

## Abstract

*Artemisia afra* Jacq. ex Willd. is a widely used medicinal plant in South Africa for the treatment of diabetes. This study aimed to evaluate the hypoglycemic activity and possible toxicity effect of aqueous leaf extract of the herb administered at different dosages for 15 days in streptozotocin-induced diabetic rats. Administration of the extract at 50, 100, and 200 mg/kg body weight significantly (*P* < 0.05) increased body weight, decreased blood glucose levels, increased glucose tolerance, and improved imbalance in lipid metabolism in diabetic rats. These are indications of antidiabetic property of *A. afra* with 200 mg/kg body weight of the extract showing the best hypoglycemic action by comparing favourably well with glibenclamide, a standard hypoglycemic drug. The extract at all dosages tested also restored liver function indices and haematological parameters to normal control levels in the diabetic rats, whereas the kidney function indices were only normalized in the diabetic animals administered with 50 mg/kg body weight of the extract. This investigation clearly showed that in addition to its hypoglycemic activity, *A. afra* may also protect the liver and blood against impairment due to diabetes. However, some kidney functions may be compromised at high dosages of the extract.

## 1. Introduction

Diabetes mellitus is a major endocrine disorder and growing health problem in most countries. It is a metabolic disease as old as mankind; and its incidence is considered high all over the world [1]. Increase in sedentary lifestyle, consumption of energy-rich diets, and obesity are some of the factors causing the rise in the number of diabetics. The World Health Organization (WHO) estimated diabetes in adults to be around 173 million, and about two-thirds of these patients live in developing countries [2]. The prevalence of diabetes is on the increase worldwide including South Africa; and it is still expected to increase by 5.4% in 2025 [3]. WHO further reports that over 4.8 million annual deaths (9% of global total) are attributable to either diabetes or its complications [4].

Despite these alarming statistics, there is no specific and definite therapy currently for diabetes. However, a few chemotherapeutic drugs have been in use to manage the disease since the accidental discovery of the hypoglycemic action of sulfonamides [5]. The thrust of such management measures is to achieve an effective blood glucose control or utilization, with a view to delaying or averting the onset of complications. The application of these measures is, however, limited due to their high cost and associated side effects. Consequently, attention is being focused on the use of herbal medicines for the treatment of diabetes.

In traditional African societies, phytotherapy is highly valued and widely utilized. South Africa, particularly, has remarkable biodiversity and rich cultural traditions of plant use. Hence, it is not surprising why the majority of the population in South Africa use plant materials as their source of primary healthcare and as an alternative or supplement to visiting western healthcare practitioners [6]. This is particularly true for the treatment of diabetes, and WHO has also authenticated phytotherapeutic approach to the treatment of the disease [7, 8]. The use of natural remedies for diabetes treatment is also strengthened due to the belief that herbs can provide some benefits over allopathic medicine and allows users to feel that they have some control in their choice of medication [9]. However, their general acceptability has been limited by lack of dose regimen and adequate data on their toxicity. Traditional medical practitioners and users of medicinal plants also believe that herbs are safe simply because they are natural in origin. It is, therefore, pertinent to provide information on the effective dose and toxicity risk associated with the use of these medicinal plants for the treatment of ailments.

*Artemisia afra* Jacq. ex Willd. (Asteraceae) is known as *Umhlonyane* in Xhosa and African wormwood in English. It is one of the widely used medicinal plants in South Africa because of its acclaimed healing properties against many ailments including diabetes. It is an erect, shrubby, and perennial plant growing up to 2 m tall with a leafy and hairy stem. The leaf shape is narrowly ovate, feathery, and finely divided, which grows up to 8 cm long and 4 cm wide. It is widespread in all the provinces of South Africa except the Northern Cape, and it is easily identifiable by its characteristic aromatic smell [6]. Despite the widespread abundance and traditional use of this indigenous plant in South Africa, no systematic study has been done to substantiate its acclaimed anti-diabetic property. The present study was designed to evaluate the antidiabetic activity and the safety/toxicity risk associated with the use of aqueous leaf extract of *A. afra* in streptozotocin-induced diabetic rats. The efficacy was compared with glibenclamide, a standard hypoglycemic drug.

## 2. Materials and Methods

### 2.1. Chemicals

Streptozotocin (STZ) was procured from Sigma Chemical Co., St. Louis, MO, USA while Glibenclamide was a product of Taj Pharmaceuticals Ltd., India. The assay kits used for biochemical assays were products of Randox Laboratories Limited, Ardmore, Co Antrim, UK. All other chemicals and reagents used were of analytical grade.

### 2.2. Plant Material and Authentication

Freshly picked *A. afra* comprising mature leaves and stems were collected from the University of Fort Hare, Alice (Eastern Cape Province), South Africa, in June 2009. The plant was authenticated by Professor D.S. Grierson, a botanist in the Department of Botany at the University of Fort Hare, and a voucher specimen (Sunmed. 2009/01) was prepared and deposited at the Giffen Herbarium of the university.

### 2.3. Preparation of Aqueous Extract

The aqueous extract of the plant was prepared in a manner that mimicked the traditional method of preparation. Briefly, the leaves of *A. afra* plucked from the stalks were rinsed with distilled water, dried in the oven at 30°C, and slightly crushed by hand. The dried leaves (100 g) were suspended in 1 L distilled water and the mixture boiled for 30 min. The decoction obtained was cooled, filtered, frozen at −70°C, and then freeze-dried (Virtis benchtop K, Virtis Company, Gardiner, NY, USA) to give a yield of 17.4 g. This was reconstituted separately in distilled water to give the required doses for the experiment.

### 2.4. Animals Used

Male albino rats of Wistar strain with a mean weight of 152 ± 5.32 g were obtained from the Experimental Animal House of the Agricultural and Rural Development Research Institute (ARDRI), University of Fort Hare, Alice, South Africa. The animals were housed in clean metabolic cages placed in a well-ventilated house with optimum condition (temperature: 23 ± 1°C; photoperiod: 12 h natural light and 12 h dark; and humidity: 45–50%). They were acclimatized to animal house conditions and allowed free access to commercial pelleted rat chow (Pioneer Foods (Pty) Ltd., Huguenot, South Africa) and water. The cleaning of the cages was done on a daily basis. This study was carried out following the approval from the Ethical Committee on the Use and Care of Animals of the University of Fort Hare, South Africa, and an ethical clearance number (AFO011) was assigned for the project.

### 2.5. Induction of Diabetes in Rats

The rats were fasted for 18 h, and diabetes was induced by a single intravenous injection of freshly prepared solution of STZ (55 mg/kg of body weight) in 0.1 M citrate buffer (pH 4.5) [10]. The animals were allowed to drink 5% glucose solution to protect them against the diabetogenic action of STZ and subsequently kept fasting in order to avoid excessive accumulation of feeding glucose which may antagonize STZ effect. Control rats were injected with citrate buffer alone. After 24 h of injection, fasting blood glucose level was checked, and animals with levels above 13.9 mmol/L were considered diabetic [10].

### 2.6. Experimental Design

The rats were divided into two sets, each comprising six groups (*n* = 6 in each group): one for antidiabetic/toxicity studies and the other for the evaluation of glucose tolerance. Group 1 (normal control) and Group 2 (diabetic control) were administered with 0.5 mL of distilled water. Groups 3 to 5 are diabetic rats treated with 50, 100, and 200 mg/kg body weight/day of *A. afra* extract, respectively, while Group 6 comprised diabetic animals administered with glibenclamide (600 *μ*g/kg body weight/day).

### 2.7. Collection of Blood Sample and Isolation of Organs

After 15 days of extract administration, the rats were humanely sacrificed by anaesthetization and the neck area was quickly cleared of fur before the jugular vein was sharply cut with sterile surgical blade. An aliquot (2 mL) of blood was collected into ethylene diamine tetra-acetic acid (EDTA) embedded sample bottles for haematological analysis. Another 5 mL of the blood was collected and centrifuged at 1282 g × 5 min, and the serum was carefully aspirated with a Pasteur pipette into sample bottles for the various biochemical assays. The rats were quickly dissected and the whole liver and two kidneys were excised, freed of fat, blotted with clean tissue paper, and then weighed. The organ-to-body weight ratio was determined by comparing the weight of each organ with the final body weight of each rat.

### 2.8. Water, Feed, Weight, and Blood Glucose Monitoring

The water intake, feed intake, and body weight gain of all the rats were monitored during the 15-day experimental period. At an interval of 5 days, blood samples were collected from the tail vein of the experimental animals after overnight fasting for the estimation of blood glucose level using a glucometer (Accu-Chek, Roche Products (Pty) Ltd., South Africa).

### 2.9. Analysis of Lipid Profile and Total Protein

The serum concentrations of total cholesterol, triglycerides, HDL-cholesterol, and LDL-cholesterol were determined by automatic analyser technique (Beckman Coulter Inc., Ireland). Total protein in the serum was estimated using bovine serum albumin as standard [11].

### 2.10. Liver and Kidney Function Tests

The concentrations of creatinine [12], urea [13], calcium [14], uric acid [15], total bilirubin [16], albumin, and globulin [17] as well as the activities of alkaline phosphatase (ALP) [18], gamma glutamyl transferase (GGT) [19], and aspartate and alanine transaminases (AST and ALT) [20] were determined in the serum using Randox Assay kits.

### 2.11. Determination of Haematological Parameters

Using Horiba ABX 80 Diagnostics (ABX Pentra Montpellier, France), the following analyses were carried out: red blood count (RBC), haemoglobin (HGB), packed cell volume (PCV), mean corpuscular volume (MCV), mean corpuscular haemoglobin (MCH), mean corpuscular haemoglobin concentration (MCHC), platelet (PLT), white blood cells (WBCs), and white blood cell differential counts.

### 2.12. Oral Glucose Tolerance Test

On day 15, the rats in groups 1 to 6 (from the second set) were given glucose (2 g/kg body weight; p.o.) 30 min after administration of the extract/drug [21]. Blood samples were collected from the tail vein prior to glucose administration and at 30, 60, and 90 min after glucose loading for immediate measurement of blood glucose levels.

### 2.13. Statistical Analysis

Data were expressed as mean ± SE of six replicates and subjected to one-way analysis of variance (ANOVA) followed by Duncan's multiple range test to determine significant differences in all the parameters. Values were considered statistically significant at *P* < 0.05.

## 3. Results

### 3.1. Water/Feed Intake and Weight Gain

While water consumption increased in the untreated diabetic rats (Group 2), the administration of aqueous extract of *A. afra* significantly reduced (*P* < 0.05) the quantity of water and feed intake in diabetic animals (Table 1). Similarly, the untreated diabetic rats showed polyphagic condition and consumed higher quantity of feed compared to the control and treatment groups. There was a significant reduction (*P* < 0.05) in the weight gained by the untreated diabetic rats when compared with the control and treatment groups. Generally, the effect of treatment with 200 mg/kg body weight of *A. afra* compared favourably well with that of glibenclamide which is a known standard drug for diabetes.

### 3.2. Blood Glucose Level

The continuous administration of aqueous extract of *A. afra* was found to significantly reduce (*P* < 0.05) the blood glucose level in diabetic rats at the end of the experiment (Table 2). Again, the effect was more pronounced in the rats treated with 200 mg/kg body weight of the extract and it compared favourably well with glibenclamide-treated rats.

### 3.3. Serum Lipid Profile and Total Protein

There was a significant elevation (*P* < 0.05) in the levels of serum cholesterol, triglycerides, and LDL and reduced HDL and protein concentrations in diabetic rats when compared with the control group (Table 3). The aqueous extract of *A. afra* and glibenclamide significantly reduced (*P* < 0.05) the levels of serum cholesterol, triglycerides, and LDL and increased HDL and protein concentration to near normalcy as observed in the control after 15 days of treatment.

### 3.4. Oral Glucose Tolerance Test

Table 4 shows the blood glucose levels of the rats after oral administration of glucose. The level in the control rats rose to the peak 30 min after glucose load and decreased to near normal levels at 90 min. In the untreated diabetic rats, the peak increase in blood glucose concentration was observed after 30 min and remained high over the next 60 min. *A. afra*- and glibenclamide-treated diabetic rats showed significant decrease (*P* < 0.05) in blood glucose concentration at 60 and 90 min compared with diabetic control rats.

### 3.5. Liver Function Parameters

The untreated diabetic rats exhibited significant increase (*P* < 0.05) in serum activities of ALP, GGT, ALT, AST, liver-to-body weight ratio, and bilirubin; as well as reduced albumin and globulin concentrations when compared with the control (Table 5). Continuous administration of aqueous extract of *A. afra* to diabetic rats for 15 days was able to restore all the liver function indices back to normalcy.

### 3.6. Kidney Function Parameters

A significant increase (*P* < 0.05) was observed in all the kidney function indices examined in the untreated diabetic rats when compared with the control (Table 6). The aqueous extract of this herb had a positive impact on the kidney function indices of diabetic rats by significantly reducing (*P* < 0.05) kidney-to-body weight ratio as well as the serum concentrations of calcium ion, creatinine, urea, and uric acid. The positive impact is, however, more pronounced in the rats treated with 50 mg/kg body weight extract.

### 3.7. Haematological Parameters

In addition, the diabetic rats exhibited significantly reduced levels (*P* < 0.05) in all the haematological parameters with the exception of white blood cell count and lymphocytes which were significantly increased (Table 7). Oral administration of aqueous extract of *A. afra* in diabetic rats for 15 days, however, restored the haematological parameters to normalcy with the exception of platelets and neutrophils which were significantly increased but not to the control levels.

## 4. Discussion

The observation of higher consumption of water and food accompanied by high blood glucose levels and urine output is an indication of diabetic state in the animals resulting from STZ administration. The present study demonstrated that aqueous extract of *Artemisia afra* has antidiabetic activity; and the efficacy is comparable to glibenclamide, a standard hypoglycemic drug.

Administration of the plant extract was effective in preventing polydipsia and polyphagia conditions. Similar observation was reported by Shetty et al. [22] using *Momordica charantia* in diabetic rats. Despite the high feed and water intake in the untreated diabetic rats, the gain in body weight was very minimal compared to the extract treated groups. The enhancement of body weight in the *A. afra*-treated rats could be attributed to the increase in metabolic activity of their body systems. This clearly indicates that the plant extract increased glucose metabolism which enhanced body weight in the rats. Again, this observation was reported by Ravi et al. [10]. According to these authors, *Eugenia jambolana* seed kernels increased body weight of diabetic rats. Of particular interest is the fact that the effect of *A. afra* at the dose of 200 mg/kg body weight compared favourably well with glibenclamide.

The increase in blood glucose concentration is an important characteristic feature of diabetic state. *A. afra* extract produced significant hypoglycemic effect on diabetic rats, and by day 15, the glucose levels tended towards normalcy as was found in the control rats. Microchemical analyses of *A. afra* have indicated the presence of saponins [23], which have been reported to possess hypoglycemic activity in diabetic rabbits [24]. Therefore, the hypoglycemic activity of *A. afra* observed in this study could be attributed to the presence of saponins which might be acting as a stimulant for the release of insulin following the repair of pancreatic beta cells by the extract [25].

Abnormalities in lipid profile are very common in the diabetic state [26]. Although lipoprotein alteration is an intrinsic part of diabetic mellitus, such alterations are also induced by diabetes-associated complications such as obesity or renal disease [27]. In the present study, aqueous extract of *A. afra* was able to bring down the levels of cholesterol, triglycerides, and LDL but increased the levels of HDL in diabetic rats to near normal levels when compared to the untreated diabetic group. The serum level of cholesterol is usually increased in diabetes, and such an elevation is a risk factor for coronary heart disease. The abnormal high concentration of cholesterol in the blood during diabetes is mainly due to the increase in the mobilization of free fatty acids from the peripheral depots, since insulin inhibits the hormone-sensitive lipase [28]. Administration of *A. afra* to diabetic rats significantly decreased the plasma cholesterol level to near normalcy and therefore reduced the risk of cardiovascular disease [29]. An increase in the concentrations of total cholesterol and LDL-cholesterol and reduced HDL-cholesterol as observed during diabetes are associated with raised risk of myocardial infarction [30]. Treatment of diabetic rats with *A. afra* extract elevated HDL-cholesterol and reduced LDL-cholesterol levels, which are indications of reduced risk of myocardial infarction. There is a growing body of evidence from epidemiologic, clinical, and laboratory data indicating that elevated triglyceride level is an independent risk factor for cardiovascular disease [31]. Hypertriglyceridemia is a characteristic condition observed in diabetics. In this study, treatment with *A. afra* extract has prevented the elevation of triglycerides, signifying that the myocardial membrane is intact and not damaged.

During diabetes, there is increased protein catabolism with flow of amino acids into the liver, which feeds gluconeogenesis [32]. These authors reported that accelerated proteolysis of uncontrolled diabetes occurs as a result of deranged glucagon-mediated regulation of cyclic AMP formation in insulin deficiency. This might have accounted for the observed decrease in the total protein content in STZ-induced untreated diabetic rats. Administration of aqueous extract of *A. afra* to diabetic rats significantly inhibited proteolysis caused by insulin deficiency and thus increased the level of plasma proteins to near normalcy.

In this study, *A. afra* extract enhanced glucose utilization by significantly reducing blood sugar level in the glucose-loaded rats. The possible mechanism by which the extract achieved this may be by increasing pancreatic secretion of insulin from beta cells of pancreas [25].

One major problem associated with the use of herbs for treating ailments is the choice of dosage. Most of the herbal preparations are administered without any standard dosage which may have some toxicological implications on vital organs in the body. The increase in liver-to-body weight ratio observed in the untreated diabetic rats in this study may be an indication of liver inflammation [33] which probably accounted for the increase in serum levels of bilirubin and marker enzymes; as well as reduced albumin and globulin concentrations.

 Alkaline phosphatase is a liver marker enzyme often employed to assess the integrity of plasma membrane and endoplasmic reticulum [34], while GGT is a membrane-localized enzyme that plays a major role in glutathione metabolism in the liver [35]. Damage to structural integrity of the liver is reflected by an increase in the activity of these two enzymes in the serum, probably as a result of leakage from altered cell membrane structure. Therefore, the increase in serum ALP and GGT activities in the untreated diabetic rats confirms damage to the plasma membrane, leading to a compromise of membranal integrity [36]. The transaminases (AST and ALT) are well-known enzymes used as biomarkers to predict possible toxicity to the liver [37]. Elevation in serum activities of both transaminases as observed in diabetic rats suggested damage to the liver cells as well [38].

Oral administration of aqueous extract of *A. afra* attenuated the elevated activities of all investigated enzymes in diabetic rats comparable to the control. This may be an indication of nontoxic nature and protective action of the extract in reversing liver damage due to diabetes. Similar observation was also reported by Ravi et al. [10] using *Eugenia jambolana* seed kernels in STZ-induced diabetic rats.

 Albumin and globulin are mixtures of protein molecules that are used to assess the health status of the liver. Albumin, which is manufactured in the liver, is a major carrier protein that circulates in the bloodstream while globulins are larger proteins responsible for immunologic responses [39]. Low serum albumin and globulin concentrations suggest chronic damage to the liver as a result of infection [40]. Therefore, the reduction in serum albumin and globulin levels in the untreated diabetic rats is an indication of diminished synthetic function of the liver. Oral administration of *A. afra* extract, however, restored the albumin and globulin levels to normalcy. This further confirmed conferment of protection to the liver of diabetic rats.

Bilirubin is the major product that results from the breakdown and destruction of old red blood cells. It is an important metabolic breakdown product of blood with biological and diagnostic values [39]. It is removed from the body by the liver; hence, it is a good indication of the health status of the liver. Elevated serum level of bilirubin in diabetic rats as observed in the present study may be a result of reduced uptake arising from liver disease. Treatment with *A. afra* extract was able to reverse this condition in diabetic rats, thereby lowering the bilirubin level to normalcy. All the data obtained with respect to liver function indices indicated absence of any significant liver damage as a result of treatment with aqueous extract of *A. afra* in diabetic rats.

As observed in the liver of untreated diabetic rats, the significant increase in kidney-to-body weight ratio may also be a result of inflammation [33]. This is an indication of kidney damage which probably accounted for the reduced functional capacity as reflected by the increased serum levels of calcium ion, creatinine, urea, and uric acid.

Glucose excretion in urine by diabetics imposes an osmotic diuresis [41], with the consequence of electrolyte loss and dehydration. An attempt by the kidney to buffer the urine decreases electrolytes such as calcium in the serum [42]. Treatment with *A. afra* extract significantly reduced the serum calcium levels when compared with untreated diabetic rats. The reduction is more pronounced in rats treated with 50 mg/kg body weight of the extract as the serum calcium level tended towards normalcy in this group. This is an indication that the extract at this dosage could restore the osmotic regulatory functions of the kidney.

The increase in serum levels of urea, creatinine, and uric acid in the untreated diabetic rats as observed in the present study is expected. Deficiency of insulin and consequent inability of glucose to reach the extrahepatic tissues stimulate gluconeogenesis as an alternative route of glucose supply [5]. This route is sustained by increased proteolysis which releases free glucogenic amino acids into the plasma that are deaminated in the liver with the consequence of increased urea in the blood. Creatinine is a metabolite of muscle creatine, and the concentration in serum is proportional to the body muscle mass. The amount of creatinine is usually constant; hence, elevated levels indicate diminished renal function only, since it is easily excreted by the kidneys [41]. Uric acid is the major metabolic product of purine metabolism, and its elevated level in the serum signifies kidney impairment. Administration of aqueous extract of *A. afra*, however, produced a significant reduction in the levels of these three metabolites, thereby conferring protection against impairment due to diabetes. Similar observations have been reported using *Picrorhiza kurroa* and *Vernonia amygdalina* extracts in diabetic rats [21, 43]. Of particular interest is the fact that the effect of *A. afra* at the dosage of 50 mg/kg body weight compared favourably well with the control.

Changes in hematological profile are very common in the diabetic state [44]. The observed reduction in the concentrations of RBC, HGB, MCH, MCHC, MCV, PCV, PLT, neutrophils, and eosinophils as well as increased concentrations of WBC and lymphocytes in the diabetic rats indicated impairment in hematological function. Administration of *A. afra* extract reversed these abnormal situations and restored normalcy. This again suggested hematoprotective ability of the extract. Similar observation was also reported with *Solanum lycocarpum* and *Phellinus igniarius* on some hematological parameters in diabetic rats [44, 45].

## 5. Conclusions

Oral administration of aqueous extract of *Artemisia afra* showed hypoglycemic activity in STZ-induced diabetes in experimental Wistar rats. The results also revealed the beneficial effects of this herb in improving the imbalance in lipid metabolism experienced during diabetes. It can, therefore, be concluded from this study that the aqueous leaf extract of *A. afra*, besides its hypoglycemic action, could protect the liver, kidney, and blood against impairment due to diabetes. However, some renal functions may be compromised at higher dosages of the extract.

## Figures and Tables

**Table 1:** Effect of oral administration of aqueous extract of *Artemisia  afra* on water intake, feed intake, and body weight in diabetic rats (*n* = 6,  mean ± SE).

Groups	Water intake (mL/day)	Feed intake (g/day)	Weight gain (g)
Control	19.08 ± 1.07^a^	14.07 ± 1.47^a^	31.30 ± 4.50^a^
Diabetic control	91.89 ± 2.13^b^	33.90 ± 3.14^b^	3.17 ± 0.96^b^
Diabetic + 50 mg/kg *A. afra *	83.84 ± 1.88^c^	27.18 ± 1.09^c^	6.76 ± 1.12^c^
Diabetic + 100 mg/kg *A. afra *	77.55 ± 1.53^d^	22.23 ± 1.06^d^	11.60 ± 1.41^d^
Diabetic + 200 mg/kg *A. afra *	44.94 ± 1.31^e^	19.03 ± 1.17^e^	19.82 ± 2.14^e^
Diabetic + glibenclamide	47.03 ± 1.47^e^	20.97 ± 1.01^e^	16.20 ± 1.17^e^

Values with different superscripts along the same column indicate statistically significant difference at *P* < 0.05.

**Table 2:** Effect of oral administration of aqueous extract of *Artemisia  afra* on blood glucose levels in diabetic rats (*n* = 6,  mean ± SE).

Groups	Blood glucose (mmol/L)			
	Initial	Day 5	Day 10	Day 15
Control	5.24 ± 0.69^a^	5.26 ± 0.72^a^	5.26 ± 0.61^a^	5.24 ± 0.85^a^
Diabetic control	5.26 ± 0.61^a^	24.90 ± 1.22^b^	27.44 ± 1.36^b^	21.60 ± 0.24^b^
Diabetic + 50 mg/kg *A*. *afra *	5.26 ± 0.72^a^	24.80 ± 1.12^b^	17.70 ± 1.22^c^	7.80 ± 0.04^c^
Diabetic + 100 mg/kg *A*. *afra *	5.24 ± 0.68^a^	24.28 ± 1.19^b^	16.70 ± 1.36^c^	7.10 ± 0.05^c^
Diabetic + 200 mg/kg *A*. *afra *	5.25 ± 0.71^a^	24.20 ± 1.00^b^	10.58 ± 1.33^d^	5.47 ± 0.26^a^
Diabetic + glibenclamide	5.25 ± 0.69^a^	24.30 ± 1.19^b^	10.70 ± 1.31^d^	5.40 ± 0.06^a^

Values with different superscripts along the same column indicate statistically significant difference at *P* < 0.05.

**Table 3:** Effect of oral administration of aqueous extract of *Artemisia  afra* on serum lipid profile and total protein in diabetic rats (*n* = 6,  mean ± SE).

Groups	Cholesterol (mg/dL)	Triglycerides (mg/dL)	HDL (mg/dL)	LDL (mg/dL)	Total protein (g/L)
Control	50.43 ± 2.70^a^	95.73 ± 2.50^a^	16.92 ± 1.98^a^	35.19 ± 2.01^a^	78.60 ± 1.69^a^
Diabetic control	76.62 ± 1.22^b^	154.27 ± 4.31^b^	10.22 ± 1.89^b^	58.10 ± 1.12^b^	63.41 ± 2.72^b^
Diabetic + 50 mg/kg *A*. *afra *	56.97 ± 2.57^a^	102.28 ± 3.80^a^	14.33 ± 0.97^a^	43.12 ± 2.80^a^	73.92 ± 2.18^a^
Diabetic + 100 mg/kg *A*. *afra *	56.85 ± 2.40^a^	101.88 ± 3.44^a^	14.28 ± 0.98^a^	42.32 ± 3.62^a^	76.92 ± 2.66^a^
Diabetic + 200 mg/kg *A*. *afra *	54.40 ± 1.22^a^	98.30 ± 3.56^a^	13.36 ± 1.00^a^	40.66 ± 3.50^a^	74.20 ± 3.84^a^
Diabetic + glibenclamide	53.95 ± 2.80^a^	96.19 ± 3.68^a^	13.01 ± 0.99^a^	38.63 ± 1.98^a^	73.08 ± 3.78^a^

Values with different superscripts along the same column indicate statistically significant difference at *P* < 0.05.

**Table 4:** Effect of oral administration of aqueous extract of *Artemisia  afra* on blood sugar levels in glucose-loaded diabetic rats (*n* = 6,  mean  ± SE).

Groups	Blood glucose (mmol/L)			
	Fasting	30 minutes	60 minutes	90 minutes
Control	4.43 ± 0.22^a^	5.87 ± 0.26^a^	5.03 ± 0.26^a^	4.53 ± 0.25^a^
Diabetic control	21.08 ± 1.26^b^	36.90 ± 1.22^b^	30.93 ± 1.28^b^	22.90 ± 1.23^b^
Diabetic + 50 mg/kg *A*. *afra *	6.10 ± 0.84^c^	15.20 ± 0.85^c^	12.10 ± 0.94^c^	9.10 ± 0.85^c^
Diabetic + 100 mg/kg *A*. *afra *	5.90 ± 0.83^c^	14.50 ± 0.83^c^	10.05 ± 0.83^c^	8.05 ± 0.84^c^
Diabetic + 200 mg/kg *A*. *afra *	5.40 ± 0.86^c^	8.67 ± 0.35^d^	7.01 ± 0.33^d^	5.07 ± 0.36^a^
Diabetic + glibenclamide	5.30 ± 0.35^c^	8.90 ± 0.34^d^	7.50 ± 0.33^d^	5.01 ± 0.33^a^

Values with different superscripts along the same column indicate statistically significant difference at *P* < 0.05.

**Table 5:** Effect of aqueous extract of *Artemisia  afra* on some liver function parameters of diabetic rats (*n* = 6 ± SE).

	Control	Diabetic	Diabetic + *Artemisia afra* (mg/kg body weight)		
			50	100	200
Liver-to-body weight ratio (%)	2.41 ± 0.19^a^	3.96 ± 0.24^b^	2.41 ± 0.20^a^	2.50 ± 0.46^a^	2.60 ± 0.18^a^
Total bilirubin (*µ*mol/L)	0.50 ± 0.07^a^	1.89 ± 0.11^b^	0.58 ± 0.03^a^	0.63 ± 0.08^a^	0.63 ± 0.07^a^
Albumin (g/L)	24.94 ± 1.22^a^	19.16 ± 0.98^b^	24.52 ± 1.22^a^	24.20 ± 1.00^a^	23.90 ± 1.27^a^
Globulin (g/L)	53.66 ± 3.40^a^	44.25 ± 0.99^b^	52.40 ± 1.80^a^	49.72 ± 1.08^a^	49.18 ± 1.07^a^
Serum alkaline phosphatase (U/L)	12.34 ± 1.70^a^	30.16 ± 2.01^b^	13.71 ± 1.20^a^	14.53 ± 1.25^a^	15.08 ± 1.80^a^
Serum *γ*-glutamyl transferase (U/L)	3.32 ± 0.02^a^	8.11 ± 0.08^b^	3.47 ± 0.06^a^	3.48 ± 0.06^a^	3.48 ± 0.08^a^
Serum alanine transaminase (U/L)	16.71 ± 1.98^a^	37.54 ± 1.23^b^	19.21 ± 2.47^a^	20.19 ± 1.90^a^	20.44 ± 1.93^a^
Serum aspartate transaminase (U/L)	11.29 ± 1.53^a^	22.30 ± 1.23^b^	11.75 ± 1.30^a^	12.62 ± 1.00^a^	13.49 ± 1.02^a^

Values carrying different superscripts from the control for each parameter are significantly different (*P* < 0.05).

**Table 6:** Effect of aqueous extract of *Artemisia  afra* on some kidney function parameters of diabetic rats (*n* = 6 ± SE).

	Control	Diabetic	Diabetic + *Artemisia afra* (mg/kg body weight)		
			50	100	200
Kidney-to-body weight ratio (%)	5.32 ± 0.35^a^	9.91 ± 0.14^b^	5.87 ± 0.46^a^	7.84 ± 0.21^c^	8.57 ± 0.19^d^
Calcium (mmol/L)	1.49 ± 0.04^a^	2.99 ± 0.08^b^	1.56 ± 0.03^a^	1.87 ± 0.05^c^	1.98 ± 0.04^c^
Creatinine (mg/dL)	31.52 ± 0.84^a^	59.25 ± 0.50^b^	33.49 ± 1.00^a^	43.34 ± 0.48^c^	47.28 ± 0.38^d^
Urea (mg/dL)	36.42 ± 3.50^a^	163.32 ± 5.31^b^	41.72 ± 1.36^a^	70.60 ± 2.08^c^	81.29 ± 2.29^d^
Uric acid (mg/dL)	5.03 ± 0.21^a^	9.74 ± 0.16^b^	5.29 ± 0.18^a^	6.92 ± 0.09^c^	6.97 ± 0.08^c^

Values carrying different superscripts from the control for each parameter are significantly different (*P* < 0.05).

**Table 7:** Effect of aqueous extract of *Artemisia  afra* on some haematological parameters of diabetic rats (*n* = 6 ± SE).

	Control	Diabetic	Diabetic + *Artemisia afra* (mg/kg body weight)		
			50	100	200
White blood cells (×10^9^/L)	7.76 ± 0.95^a^	13.15 ± 1.04^b^	8.62 ± 0.67^a^	8.71 ± 0.99^a^	8.79 ± 0.90^a^
Red blood cells (×10^12^/L)	8.28 ± 0.27^a^	6.56 ± 0.10^b^	8.07 ± 0.37^a^	7.94 ± 0.37^a^	7.92 ± 0.26^a^
Haemoglobin (g/dL)	15.43 ± 0.29^a^	12.57 ± 0.35^b^	15.35 ± 0.45^a^	15.30 ± 0.42^a^	15.08 ± 0.46^a^
Packed cell volume (L/L)	0.48 ± 0.02^a^	0.24 ± 0.02^b^	0.47 ± 0.02^a^	0.46 ± 0.03^a^	0.46 ± 0.02^a^
Mean corpuscular volume (fL)	62.43 ± 1.09^a^	53.55 ± 1.24^b^	60.18 ± 1.22^a^	59.90 ± 1.53^a^	59.85 ± 1.54^a^
Mean corpuscular haemoglobin (pg)	19.70 ± 0.65^a^	15.03 ± 0.62^b^	19.27 ± 0.46^a^	19.10 ± 0.26^a^	19.00 ± 0.41^a^
Mean corp. haemoglobin conc. (g/dL)	33.20 ± 0.51^a^	26.77 ± 0.21^b^	32.30 ± 0.59^a^	32.28 ± 0.61^a^	32.13 ± 0.58^a^
Platelets (×10^9^/L)	924.00 ± 11.36^a^	639.75 ± 12.84^b^	765.75 ± 14.10^c^	746.33 ± 15.77^c^	746.00 ± 13.16^c^
Neutrophils (%)	13.20 ± 0.28^a^	4.53 ± 0.22^b^	8.10 ± 0.41^c^	7.85 ± 0.35^c^	7.85 ± 0.31^c^
Lymphocytes (%)	60.13 ± 1.93^a^	68.67 ± 1.08^b^	61.33 ± 1.44^a^	62.34 ± 1.46^a^	62.45 ± 1.25^a^
Eosinophils (%)	3.03 ± 0.50^a^	1.15 ± 0.18^b^	2.67 ± 0.46^a^	2.50 ± 0.18^a^	2.47 ± 0.32^a^

Values carrying different superscripts from the control for each parameter are significantly different (*P* < 0.05).
